# Supplementary material for: Global hypo-methylation in a proportion of glioblastoma enriched for an astrocytic signature is associated with increased invasion and altered immune landscape
Source: eLife. 2022 Nov 22;11:e77335. doi: 10.7554/eLife.77335 (PMC9681209; doi:10.7554/eLife.77335)
Supplement: Figure 2—source data 1. [file elife-77335-fig2-data1.zip › Figure_2_source_data_1/Figure_2A/homerResults/motif54.similar.html]

motif54

## Information for motif54

A
C
T
G
A
C
T
G
A
G
T
C
A
C
T
G
A
C
T
G
A
G
C
T
A
C
G
T
A
G
T
C
C
G
T
A
G
T
A
C
  
Reverse Opposite:  

A
C
T
G
A
C
G
T
A
C
T
G
G
T
C
A
C
T
G
A
T
A
G
C
G
T
A
C
A
C
T
G
A
G
T
C
A
G
T
C
  

|  |  |
| --- | --- |
| p-value: | 1e-12 |
| log p-value: | -2.808e+01 |
| Information Content per bp: | 1.872 |
| Number of Target Sequences with motif | 16.0 |
| Percentage of Target Sequences with motif | 0.69% |
| Number of Background Sequences with motif | 3.0 |
| Percentage of Background Sequences with motif | 0.08% |
| Average Position of motif in Targets | 84.2 +/- 53.9bp |
| Average Position of motif in Background | 83.4 +/- 16.2bp |
| Strand Bias (log2 ratio + to - strand density) | 0.7 |
| Multiplicity (# of sites on avg that occur together) | 1.00 |
| Motif File: | file (matrix) reverse opposite |

### Similar de novo motifs found

|  |  |  |  |  |  |  |  |
| --- | --- | --- | --- | --- | --- | --- | --- |
| Rank | Match Score | Redundant Motif | P-value | log P-value | % of Targets | % of Background | Motif file |
